# Electromagnetic Properties of Steel Fibres for Use in Cementitious Composites, Fibre Detection and Non-Destructive Testing

**DOI:** 10.3390/ma14092131

**Published:** 2021-04-22

**Authors:** Karel Künzel, Václav Papež, Kristýna Carrera, Petr Konrád, Michal Mára, Přemysl Kheml, Radoslav Sovják

**Affiliations:** 1 Department of Electrotechnology, Faculty of Electrical Engineering, Czech Technical University in Prague, Technická 2, 166 27 Prague 6, Czech Republic; kuenzel@fel.cvut.cz (K.K.); papez@fel.cvut.cz (V.P.); 2 Experimental Centre, Faculty of Civil Engineering, Czech Technical University in Prague, Thákurova 7, 166 29 Prague 6, Czech Republic; kristyna.carrera@fsv.cvut.cz (K.C.); petr.konrad@fsv.cvut.cz (P.K.); michal.mara@fsv.cvut.cz (M.M.); premysl.kheml@fsv.cvut.cz (P.K.)

**Keywords:** cementitious composite, concrete, magnetic field, fibre, orientation, UHPFRC, quality factor

## Abstract

This paper deals with the description, measurement, and use of electromagnetic properties of ferromagnetic fibres used as dispersed fibre reinforcement in composite mixtures. Firstly, the fibres’ magnetic properties are shown, and a method of measuring the hysteresis loop of fibres is proposed. The results from the measurements are presented and a discussion of the influence of measured parameters on the fibres’ orientation in a magnetic field is performed. Furthermore, methods of non-destructive estimation, of their amount and orientation in the composite specimens, are discussed. The main experimental goal of this paper is to show the relationship between this non-destructive method’s results and the destructive flexural strength measurements. The method is sensitive enough to provide information related to fibre reinforcement.

## 1. Introduction

One of the disadvantages of concrete is its brittleness, which can be significantly suppressed by using fibres as dispersed reinforcements. Concrete together with fibres creates a composite material of high quality for a wide range of applications. Steel fibres are the most commonly used kind of fibres due to their high tensile strength and thermal expansion, similar to concrete. Many previous studies have examined the mechanical behaviour of these composites [1,2,3,4,5,6,7,8,9]. Among other things, fibre reinforcement improves ductility, controls the formation and propagation of cracks, and drastically improves energy dissipating capacity. When coupled with a suitable cementitious matrix, the composite can exhibit strain hardening and deformation hardening behaviour, so the overall strengths can also be improved. Fibres’ orientation in cementitious composites has a significant effect on their final mechanical properties [10]. Fibres are used most effectively when their orientation is in the direction of tensile stresses. Orientation in the cementitious composite, however, depends on many factors, mainly the casting method, mould shape, vibration, type of fibres, and the fresh mixture’s rheological properties. Many authors [11,12,13,14] studied how the method of casting and vibrating may affect a fibre’s orientation. Nevertheless, it is impossible to have full control over the orientation of fibres only using the methods of casting and flow. Therefore, a magnetic field can be used to achieve the desired fibre orientation. Fibres are aligned in the direction of magnetic field lines by exposing the fibre-reinforced concrete to the magnetic field [15]. It is important to mention that the magnetic field has to be strong enough to overcome the matrix’s resistance.

Before this magnetic orientation can be successfully employed, the magnetic properties of commercially available fibres should be known to properly design the process and understand the behaviour of fibres during orientation. The final moment force effects acting on a fibre depend not only on the strength of the magnetic field but also on the fibre’s magnetic properties. Moreover, the results of this process should be analysed, at least to confirm that the orientation inside the composite was achieved. This can be performed using destructive testing to compare resulting mechanical properties. However, thanks to fibres’ magnetic properties, a non-destructive method can also be used, which is the main topic of this paper.

In the first part, this paper deals with measuring the magnetic properties of different steel fibres and their effect on fibre orientation in a magnetic field. In the second part of this paper, a new non-destructive method to estimate steel fibres’ orientation is shown and tested. This non-destructive method uses a new principle based on the measurement of a quality factor of a coil, unlike the measurements of inductance [16,17,18,19,20,21]. It is shown in this paper that, with a properly designed coil, quality factor measurements are more sensitive to changes in fibre reinforcement parameters compared to inductance measurements. This method is sensitive to both fibre volume and fibre orientation. The method could be used in combination with magnetic orientation as a tool for production quality assurance.

## 2. Technical Background

The materials of fibres for application in composites are mainly chosen so that the mechanical properties of the resulting composite material are maximized. Fibres influence the tensile strength and also the ductility of the resulting specimen. According to fibre datasheets, they are made of hard, many times rolled steel to increase strength. Manufacturers guarantee dimensions, strength, flexibility, and corrosion resistance, but magnetic properties are not guaranteed nor specified in any way. Based on the chemical composition given in the datasheets, fibres are probably made from magnetically soft materials with a relatively wide hysteresis loop, similar to structural or high-strength steels. To use a fibre’s magnetic properties for its orientation or identification, it is necessary to obtain an overview of these properties.

### 2.1. Mechanical Moment Acting on a Fibre

If we assume that the magnetic field into which the fibre is inserted is homogeneous, and the fibre is so small that its presence does not affect the field, the mechanical moment acting on the fibre can be expressed as:

(1) $$\vec{M}=\vec{m}\times \vec{B},$$

where $\vec{M}$ is the vector of the mechanical moment (Nm), $\vec{m}$ is the vector of the magnetic moment (Am^2^), and $\vec{B}$ is the magnetic field induction vector (T). If *α* is the angle between the vectors $\vec{m}$ and $\vec{B}$, then the absolute values of the moment can be calculated as

(2) $$\left|M\right|=\left|m\right|\cdot \left|B\right|\cdot \sin\alpha .$$

If the fibre’s magnetic moment is constant (the fibre would act as a permanent magnet), then the mechanical moment acting on the fibre will be the most significant; if its magnetic moment vector is perpendicular to the magnetic field induction vector. The mechanical moment does not act on the fibre if the vector of its magnetic moment is parallel to the magnetic field induction vector.

If it is possible to move the fibre in the environment, the result of the mechanical moment’s action is the fibre’s rotation along its axis, given by the mechanical moment vector and the fibre’s position, where the magnetic moment vector is parallel with the magnetic field induction. The magnetic moment vector of the fibre can be expressed as

(3) $$\vec{m}=\vec{l}\cdot \;\Phi_{m},$$

where $\vec{l}$ is the length of the fibre (m) and *Φ_m_* is the magnetic charge of the dipole of the magnetic flux at the fibre’s ends (Wb).

This expression is based on the fibre’s magnetostatic representation by a magnetic dipole, formed by two opposite magnetic charges (poles) at a certain distance. The magnetic charge of the dipole is

(4) $$\Phi_{m}=S\cdot B_{n},$$

where *S* is the area of the perpendicular section of the fibre (m^2^) and *B_n_* is the normal component of the magnetic induction vector on this surface (T). The value of *B_n_*, for a fibre placed in a field with intensity *H* (Am^−1^), can be determined according to the fibre’s material magnetization characteristics as

(5) $$B_{n}=f_{B}\left(H_{n}\right),$$

where *H_n_* stands for the normal component of the magnetic field’s intensity, perpendicular to the fibre’s surface section; in other words, the projection of the vector $\vec{H}$ in the fibre’s axis. Angle *α* is also an angle between $\vec{H}$ and $\vec{l}$. We can then write:

(6) $$H_{n}=\left|H\right|\cos\alpha ,$$

and

(7) $$\vec{M}=\vec{m}\times \vec{B}=\left|m\right|\cdot \left|B\right|\cdot \sin\alpha =S\cdot l\cdot f_{B}\left(\left|H\right|\cos\alpha \right)\cdot \left|B\right|\cdot \sin\alpha .$$

We may simplify Equation (7) by approximating the magnetization characteristic by a direct ratio:

(8) B=μH,

where *µ* is the permeability, and thus obtain:

(9) $$\vec{M}=S\cdot l\cdot \mu {\left|B\right|}^{2}\cos\alpha \cdot \sin\alpha =\frac{1}{2}S\cdot l\cdot \mu {\left|B\right|}^{2}\sin2\alpha \;.$$

The size of the mechanical moment acquires its maximum value at the fibre axis’s deviation 45° from the direction of the magnetic field lines (assuming a linear approximation applies at a deviation close to this value for the nonlinear magnetization characteristic). If the fibre axis is perpendicular to the magnetic field line’s direction, or is parallel to it, torque does not occur. The mechanical moment’s value is proportional to the square of *B* if a linear approximation applies, and proportional to *B* if the fibre’s magnetic material is saturated.

### 2.2. Summary

When studying the effect of a magnetic field on a ferromagnetic fibre, we can consider the following simplified cases.

Simplification one—unsaturated state, approximately linear magnetization. As the magnetic intensity *H* of the magnetic field into which the fibre is inserted increases, the magnetic induction *B* in the fibre increases linearly. The moment acting on the fibre is proportional to the square of the magnetic field strength. During the fibre’s rotation on the magnetic system’s axis, the moment increases at first, but then the effect of the decreasing magnetic induction in the fibre prevails, and the moment acting on the fibre decreases to zero as, at that point, the fibre reaches a position perpendicular to the axis of the magnetic system.

Simplification two—saturated fibre. Assuming the fibre is already magnetized, the magnetic induction inside the fibre practically no longer grows, and the fibre behaves like a permanent magnet. The moment acting on the fibre is proportional to the intensity of the magnetic field. As the fibre rotates, it grows at an angle, reaching a maximum at the position perpendicular to the magnetic field lines’ direction.

In the real case, we must consider the transition between the unsaturated and saturated state, which respects the magnetization characteristic’s shape. Consider the function of the size of the fibre’s moment as dependant on the fibre’s angle relation to the magnetic system’s axis. Thus, the moment acting on the fibre decreases practically to zero in the vertical position. This phenomenon could also be seen in the ANSYS program’s (ANSYS Workbench, 2020 R1, ANSYS Inc., Canonsburg, PA, USA) simulation results in the next part of this article.

## 3. Measurements of the Magnetic Properties of Fibres

A measuring device was constructed to measure the magnetization curve and the fibre’s hysteresis curve, having the fibre’s unknown magnetic properties in mind. The goal of this part was to obtain the magnetic properties of ferromagnetic fibres that influence the forces acting on the fibres in the specific circumstances of the fibre orientation process. From this point of view, the maximum achievable induction in a fibre is of importance, as saturation can be achieved in a sufficiently strong magnetic field. Another parameter was the magnetisation curve’s shape, which would be related to a significant lowering of the forces acting on a non-magnetised fibre because of low initial magnetisation.

Different commercially available fibres should be subjected to this measurement, without additional changes, so as to not modify their magnetic properties. The magnetic parameters of the air-cored coil, to which the cementitious composite with fibres was inserted, had a sinusoidal shape of the magnetic intensity *H*. Because of these reasons, the standard measurements described in the family of norms IEC 60404 were not suitable. Instead, a measuring device was created to allow for the measurement of small samples, made of several fibres, to obtain a sufficiently strong measured signal. These samples were subjected to a sinusoidal shape of magnetic intensity. The measuring was based on the standard measurement of a hysteresis curve from the evaluation intensity of the magnetic field *H* and the magnetic induction *B* in the monitored material. In this case, the measuring station was modified so that it was possible to measure samples of the commonly used small ferromagnetic fibres directly. Their diameter ranges are in the order of tenths of a millimetre, and their length are in the order of millimetres.

**Figure 1:**
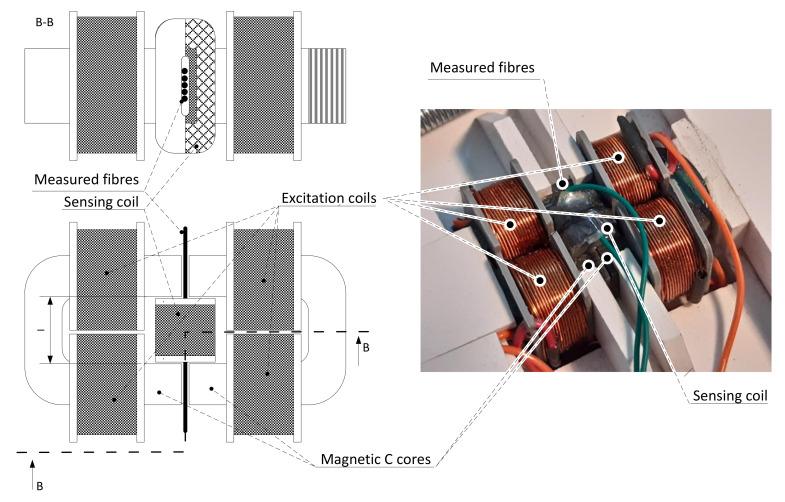
The device for measuring the magnetic properties of ferromagnetic fibres.

The measuring set’s essential elements were two C-shaped cores (Figure 1) composed of 0.18 mm thick sheets of high-quality ferromagnetic material C22 with a narrow hysteresis curve, intended for chokes and transformers. The core’s material had low hystereses loss and eddy current loss, and low magnetic resistance, which could be practically neglected compared to the measured fibre’s magnetic resistance. Four coils were mounted on these cores, creating a magnetic field with the same orientation in both C-cores. Fibre samples were sandwiched between the two C-cores. Because the magnetic induction in a single fibre would be challenging to detect, several juxtaposed fibres were always used. A specimen was prepared and pre-glued for easier handling. The fibre ends adjacent to the C-cores were cleaned to minimize additional magnetic resistance. A measuring coil surrounded the fibre sample, with a high number of turns designed to evaluate the magnetic induction in the specimen. The intensity of the magnetic field was formed by two parallel pairs of coils (each pair with one C-core), and acted on the specimen at a length which corresponded to the C-core’s inner dimension. The cross-section of a fibre was significantly smaller compared to other parts of the proposed circuit. The evaluation of the intensity of the magnetic field was obtained from the relation:

(10) $$\left|\vec{H}\right|=\frac{2N_{1\;}I_{1}}{l},$$

where *H* is the intensity of the magnetic field (Am^−1^); *l* is the active length of the specimen (the dimension of C-core gap) (m); *N*_l_ is the number of turns of the excitation coils; and *I*_1_ is the current in the excitation coils (A). The second relation is the law of induction:

(11) $$U_{2}=-N_{2\;}n_{f\;}S_{f\;}\frac{dB}{dt},$$

where *U*_2_ is the induced voltage in the sensing coil (V); *n_f_* is the number of fibres in a specimen; *S_f_* is the cross-section of an area of one fibre (m^2^); *N*_2_ is the number of turns of the sensing coil; and *B* is the magnetic induction in the specimen (T). The designed magnetic induction *B_f_* in one fibre is then given as

(12) $$B_{f}=\frac{B}{n_{f}}=\int \frac{-U_{2}}{N_{2\;}S_{f}}dt\;.$$

Reliable and sufficiently accurate evaluation of magnetic induction was performed by an integrator using a high-quality, low-noise, and low distortion operational amplifier LT1115. The device’s arrangement is shown in Figure 1. On the left, there is a schematic, and the right part of Figure 1 is a photograph of the actual design. The active parts of the product were:

- Two pcs of C core (wound by magnetic band M110-18S thickness 0.18 mm, material C22);
- Four pcs of excitation coil (112 turns of isolated Cu wire, diameter 0.46 mm), 3D printed frame;
- One pc of sensing coil (1500 turns of isolated Cu wire diameter 0.08 mm), 3D printed frame;
- Non-magnetic support and pressure mechanism.

**Figure 2:**
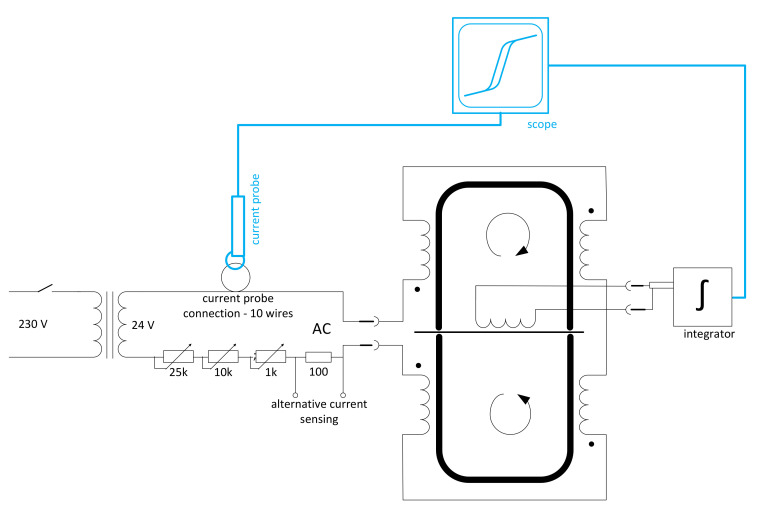
Diagram of a measuring device for determining the magnetic properties of fibres.

The overall measurement schematic is in Figure 2. The source of the excitation current was measured by a current probe. To increase the sensitivity, the current was measured on ten turns of the power conductor. The sensing coil’s output voltage was integrated and, together with the input current, was used as an input signal for a digital oscilloscope (Keysight DSOX2022A, Santa Rosa, CA, USA) operating in XY mode.

### 3.1. Results of Magnetic Measurement Properties of Fibres

The measured values obtained from the oscilloscope were processed according to the formulas given above. The hysteresis curves and magnetization characteristics of the measured materials were obtained. At first, the empty coil was measured to correct the scattering flow, which cannot be otherwise ruled out in such a small and compact arrangement.

**Figure 3:**
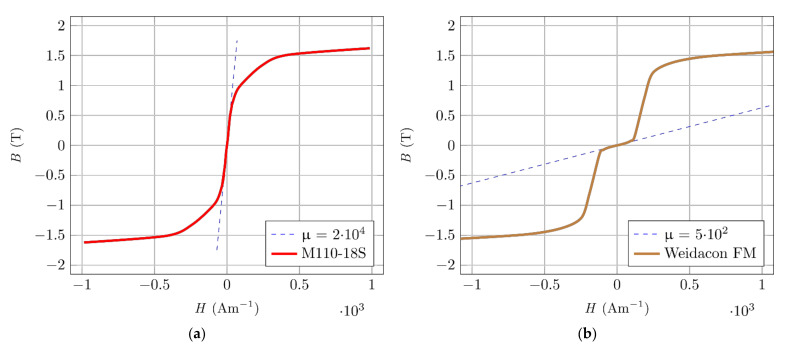
Measured magnetic characteristics. (**a**) Oriented silicon steel M110-185, C22. (**b**) Fibre Wiedacon FM.

The magnetization curves were obtained as the endpoints of the partial hysteresis curves during the excitation current change. The results were verified on known materials. For example, in Figure 3a, the magnetization characteristic of an oriented transformer sheet with a thickness of 0.18 mm can be seen (measured in the oriented direction). In Figure 3b, one can see a typical magnetization characteristic of ferromagnetic fibres (in this case, Weidacon FM). It is clear from the graph that the fibres are not primarily designed as a material with ideal magnetic properties. The material’s initial permeability is relatively low; hence, the force acting on such fibres in a weak magnetic field is not significant. The mechanical moment acting on such a fibre inserted into the magnetic field would not be sufficient for its successful orientation. If the magnetic field has sufficient intensity, the limits of the initial magnetization are exceeded, and the magnetic induction in the fibre rapidly increases to values comparable to “magnetically high-quality material”.

**Figure 4:**
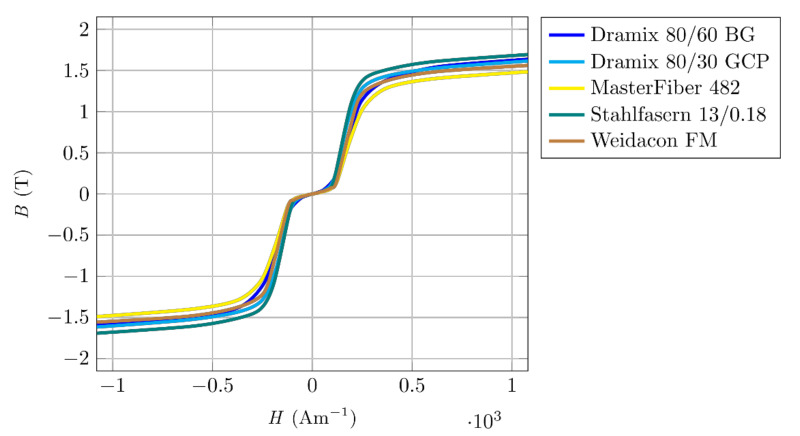
Magnetizing characteristics of selected ferromagnetic fibres.

It is clear from Figure 4 that other ferromagnetic fibres used in concrete mixes behave similarly. From the measured results, and the fact that the mechanical moment acting on the fibre in the magnetic field depends on the achieved magnetic induction in the fibre material, we can draw the following conclusions.

- If the magnetic field strength is insufficient, there is a risk that the magnetic induction required to derive the required torque would not be achieved with the demagnetized fibre.
- The magnetization characteristic and the subsequent force acting on the fibre essentially eliminates the gradual possibility of an increase of the mechanical moment. Instead, we should expect a steep growth after a certain limit is exceeded. With the variable mechanical resistance depending on the concrete mixture’s actual rheological properties, it almost eliminates the possibility of targeted partial orientation by changing the magnetic field.

**Figure 5:**
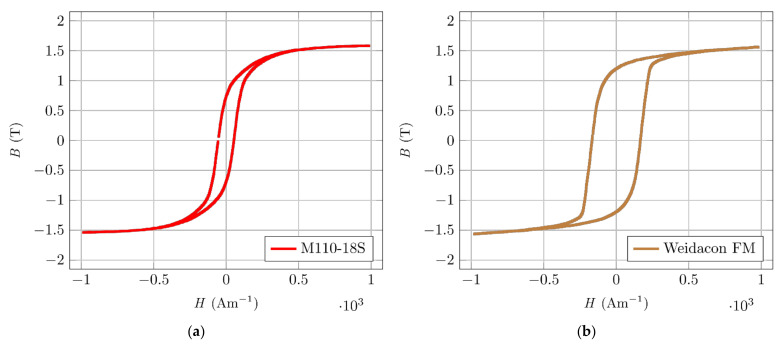
Magnetic hysteresis. (**a**) Oriented silicon steel M110-18S, C22. (**b**) Fibre Weidacon FM.

Hysteresis curves were measured using the same method during constant excitation current amplitude, at a frequency of 50 Hz (Figure 5). Comparing the ferromagnetic fibre’s typical hysteresis curve (Figure 5a) with the magnetic material for the transformer core (Figure 5b), a wider hysteresis curve is evident, meaning higher hysteresis loss of the material. The fibre’s magnetic orientation is affected only in connection with the already mentioned beginning of the magnetization characteristics. However, this can be used to detect a fibre’s presence and orientation in a specimen. A higher number of fibres and their orientation in the direction of the measuring coil axis would mean higher losses that affect the measuring coil’s magnetic circuit properties, which will be further explained later.

**Figure 6:**
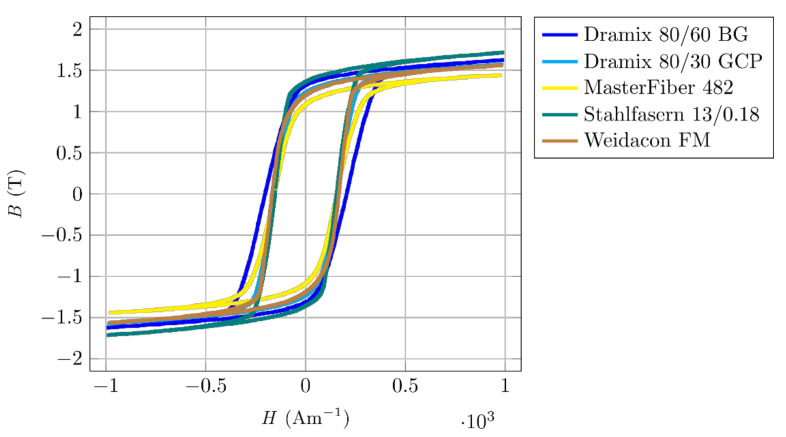
Magnetic hysteresis of selected ferromagnetic fibres.

Comparing the hysteresis curves of different ferromagnetic fibres (Figure 6) confirms a wider hysteresis curve. Different materials have a slightly different width and slope of hysteresis curve and value of magnetic remanence. However, the differences are not large and would not cause significant differences in the induced mechanical moment during magnetic orientation.

### 3.2. Simulation of the Moment Acting on a Fibre in a Magnetic Field

**Figure 7:**
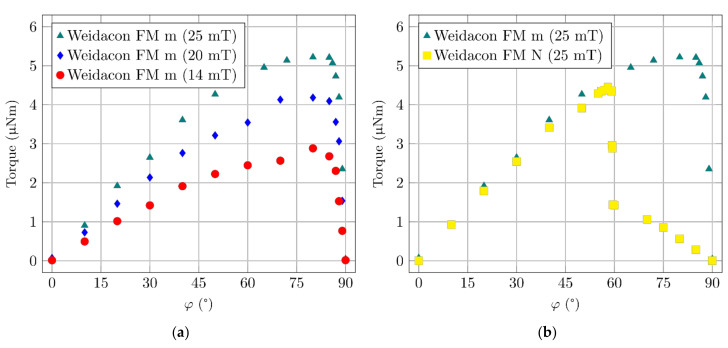
Simulation of the moment acting on the fibres depending on the angle of the fibre rotation. Fibres magnetized (m) and non-magnetized (N). (**a**) Different magnetic field intensities. (**b**) Different magnetization characteristics of the same fibre.

The measured magnetization curves were used for correct material identification in the simulations. The dependency of the moment acting on a ferromagnetic fibre on the angle between fibre and magnetic field lines was simulated in ANSYS (ANSYS Workbench, 2020 R1, ANSYS Inc., Canonsburg, PA, USA). In Figure 7a, one can see the dependence of the moment on the fibre’s angle during rotation, with respect to the magnetic system axis. The magnetic moment is following the assumptions given in Section 2. The different intensities of the magnetic field, with the same geometric arrangement, are given in Figure 7a. Figure 7b shows the magnetic moment for different magnetization characteristics for the same fibre. The graph shows the influence of nonlinear magnetization characteristics, which causes a significant decrease in torque at low intensity of the magnetic field and, thus, the fibre’s inability to overcome the matrix’s mechanical resistance in case of a non-magnetized state.

A rapid increase in magnetic induction in the fibre for smaller angles is evident from the graph in Figure 7a. Hence, a transition to a saturated state occurs, the fibre reaches a steeper part of the magnetization curve and, from a certain angle, behaves again in a practically “magnetized” manner.

### 3.3. Summary

The device was able to measure the magnetization characteristics and hysteresis curves of ferromagnetic fibres. The fibres are commonly used as dispersed reinforcement in cement composites. The results showed that different fibre materials have very similar magnetic properties; in particular, the shape and basic parameters of the hysteresis curve. However, the shape and values of the magnetization characteristics are also similar. The values of saturation of magnetic induction correspond to the basic material—structural steel. The hysteresis curve is significantly wider than that of magnetic materials for magnetic circuits of electrical machines. The wider hysteresis curve is related to the function of the magnetization characteristic, which shows an area of small relative permeability for the low intensity of the magnetic field, and the increase occurs only at higher values. In this area, the fibres behave as magnetic materials with low permeability. Magnetic induction is relatively small, which reduces the force effects on such fibre in the magnetic field. If the fibre was previously magnetized, this phenomenon could be avoided due to the relatively large remanent magnetic induction.

The measured characteristics were used in simulations of the moment acting on the fibre in a magnetic field. The simulations confirmed the expectations. The simulations further confirmed the moment reduction of non-magnetized fibres for small magnetic field strength values.

## 4. Using the Electromagnetic Properties for Estimating the Fibre Volume Content and Orientation

Concrete is a diamagnetic material, meaning the effect of water is negligible; however, steel fibres are a ferromagnetic material, which has a significant impact on the behaviour of this composite in the magnetic field. Placing such a composite into an air coil causes a decrease in magnetic resistance and an increase in the coil’s inductivity. The coil’s inductivity increases with higher volume and better orientation of fibres in the direction of magnetic field lines.

These changes of magnetic resistance and inductivity are more significant in the state where fibres are not magnetically saturated. However, it is possible to measure this phenomenon. The larger part of the space in the magnetic circuit is still air and, therefore, no differences between the different fibres’ positions and volume are observed.

If the concrete specimen is inserted into a coil that is powered by a proper current of suitable frequency, the most significant losses (mainly hysteresis losses) are induced when the fibres are correctly oriented and are present in the specimen in a high volume. Therefore, it is much more appropriate to measure the loss factor, or the quality factor *Q*, of the coil, which is the inverse value of the loss factor.

### 4.1. Quality Factor of an Air-Core Coil

The quality factor is defined as

(13) $$Q=\frac{\omega L_{s}}{R_{s}},$$

where *ωL_S_* is the imaginary part of the coil’s equivalent impedance (Ω); *R*_S_ is the real part of the coil’s equivalent impedance (Ω); and *ω* is the angular frequency (s^−1^). The frequency dependence of the quality factor is determined by the frequency dependence of the series’ equivalent resistance of the coil, because the frequency dependence of the inductance of the coil is very small. The series of the coil’s equivalent resistance corresponds to the commonly considered DC (direct current) resistance of the conductor on which the coil is wound. This is only valid in the region of very low frequencies.

An electric field is generated by induction from the passing of AC (alternating current) through a conductor. This electric field induces a current density which compensates the original current density inside the conductor and increases the original current density at the surface of the conductor. The current density *J* (Am^−2^) inside the conductor is not constant and decreases exponentially from maximum *J*_0_ on the surface to the value at depth *d*

(14) $$J\left(d\right)=J_{0}\;e^{-\frac{d}{\delta }},$$

where *δ* can be expressed as

(15) $$\delta =\sqrt{\frac{2}{\omega \mu \sigma }}\;,$$

where *μ* is the permeability of the material (Hm^−1^) and *σ* is the conductivity of the material (Sm^−1^). The consequence of the current concentration in only a part of the conductor cross-section also leads to an increase in its alternating resistance *R*(*ω*), in comparison with the direct current resistance *R*_0_. Their interdependence can be expressed as

(16) $$R\left(\omega \right)\approx R_{0\;}\left(1+D\frac{\sqrt{2}}{8}\sqrt{\omega \mu \sigma }\right).$$

The alternating resistance of the conductor forming the coil winding increases its value due to the negative effect of the interaction between the current field of one conductor and the magnetic field of the whole coil (all wires). Since the theoretical analysis of this influence is practically impossible, a mathematical approximation by a third-order polynomial is used to describe it

(17) $$R\left(\omega \right)\approx R_{0}\left(1+D\frac{\sqrt{2}}{8}\sqrt{\omega \mu \sigma }+a\omega +b\omega^{2}+c\omega^{3}\right).$$

**Figure 8:**
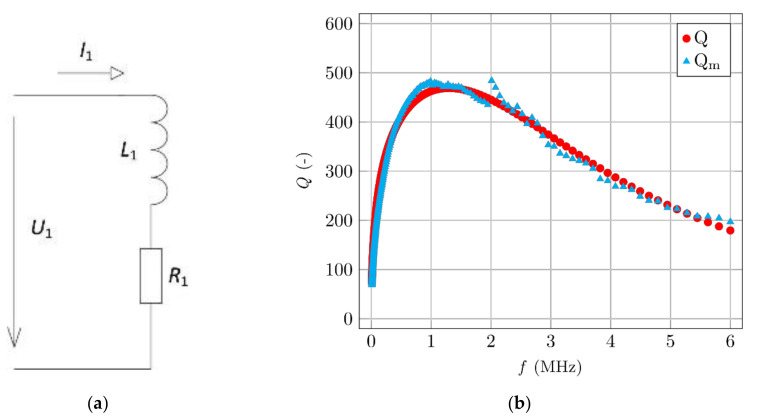
(**a**) Equivalent circuit of an air coil (*L*_1_ = *L_s_*; *R*_1_ = *R_s_*). (**b**) Air coil’s measured and calculated *Q* factors.

The values of constants *a*, *b*, *c*, can be found such that the approximate frequency dependency of the coil’s quality factor, in a wide frequency range, corresponds to the measured values. An example of the measured dependency (*Q_m_*) and the calculation (*Q*) for the measuring coil (described in the following text) is shown in Figure 8.

### 4.2. The Quality Factor of a Coil with Inserted Specimen

**Figure 9:**
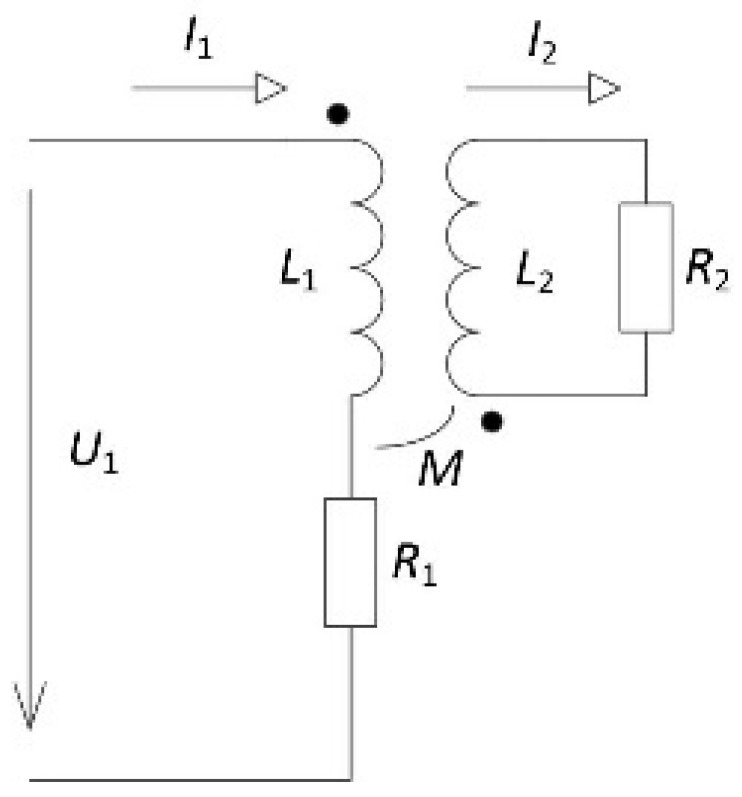
Equivalent circuit of the coil with the test specimen.

The coil’s equivalent resistance is changed by inserting a specimen with ferromagnetic fibres. It is possible to represent each fibre, or all same fibres together, by a closed LR circuit (*L*_2_, *R*_2_), which is inductively connected to the measured coil (Figure 9).

This circuit can be simplified by an ideal LR circuit with parameters *L_i_* and *R_i_*. We can then write

(18) $$Z_{i}=\frac{U_{1}}{I_{1}}=\left(R_{1}+p^{2}R_{2}\right)+j\omega \left(L_{1}+p^{2}L_{2}\right)=R_{i}+j\omega L_{i},$$

where

(19) $$p^{2}=\frac{\omega^{2}M^{2}}{R_{2}{}^{2}+\omega^{2}L_{2}{}^{2}}.$$

The equivalent resistance consists of the above approximated empty coil’s resistance and the recalculated resistance representing the inserted specimen. So, we can write

(20) $$R_{i}\left(\omega \right)=R_{1}+p^{2}R_{2},$$

and

(21) $$R_{i}=R_{0}\left(1+D\frac{\sqrt{2}}{8}\sqrt{\omega \mu \sigma }+a\omega +b\omega^{2}+c\omega^{3}\right)\;+\frac{k_{1}M^{2}\omega^{2}\sqrt{\omega }+\frac{8\pi M^{2}}{\sigma l}\omega^{2}}{L_{2}{}^{2}\omega^{2}+k_{1}{}^{2}M^{4}\omega +2k_{1}M^{2}\sqrt{\omega }+{\left(\frac{8\pi }{\sigma l}\right)}^{2}}.$$

According to theoretical assumptions, the inverse value of the resulting quality factor is equal to the sum of the inverse values of the quality factor of the empty coil and the quality factor of the ideal coil with the inserted specimen

(22) $$\frac{1}{Q_{m}\left(\omega \right)}=\frac{R_{i}\left(\omega \right)}{\omega L_{1}}=\frac{R_{1}}{\omega L_{1}}+\frac{p^{2}R_{2}}{\omega L_{1}}=\frac{1}{Q_{1}}+\frac{1}{Q_{2}},$$

which gives us the final equation

(23) $$\frac{1}{Q_{m}\left(\omega \right)}=\frac{1}{\omega L_{1}}R_{0}\left(1+D\frac{\sqrt{2}}{8}\sqrt{\omega \mu \sigma }+a\omega +b\omega^{2}+c\omega^{3}\right)\;+\frac{1}{{\omega L}_{1}}\frac{k_{1}M^{2}\omega^{2}\sqrt{\omega }+\frac{{8\pi M}^{2}}{\sigma l}\omega^{2}}{L_{2}{}^{2}\omega^{2}{+k}_{1}{}^{2}M^{4}{\omega +2k}_{1}M^{2}\sqrt{\omega \;}+{\left(\frac{8\pi }{\sigma l}\right)}^{2}}.$$

**Figure 10:**
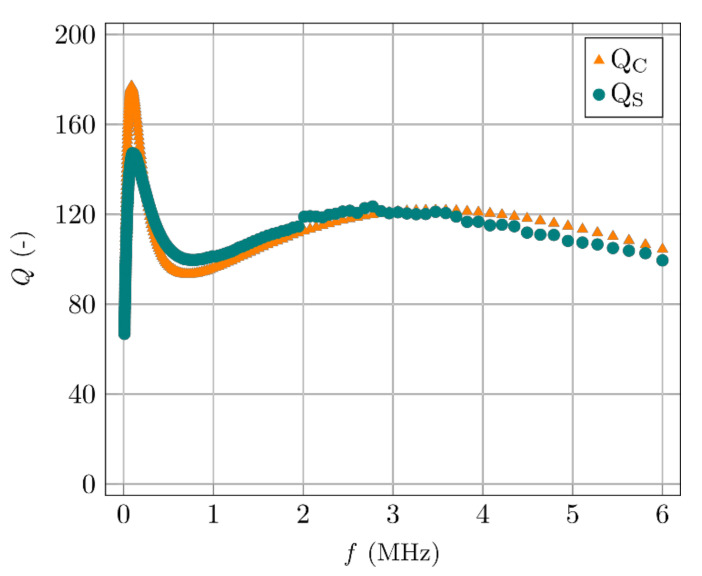
The measured coil with a concrete specimen (*Q*s) and its calculation (*Q*c).

Although the resulting formula is relatively complicated, it allows for the fitting of parameters representing the specimen to achieve compliance with the measurement (Figure 10).

### 4.3. Experiments—Description of Measurements

A series of measurements of the quality factor was performed on specimens with oriented fibres and reference specimens with non-orientated fibres. The results were compared with flexural strengths and CT scans. Two different sizes of specimens were used for the tests—smaller, with the dimensions of 40 mm × 40 mm × 160 mm, and larger, with the dimensions of 100 mm × 100 mm × 400 mm. The reference specimens (non-oriented) were made by the standard method of casting into steel moulds. The oriented specimens were cast in a plastic mould and exposed to a magnetic field to provide the desired orientation.

**Figure 11:**
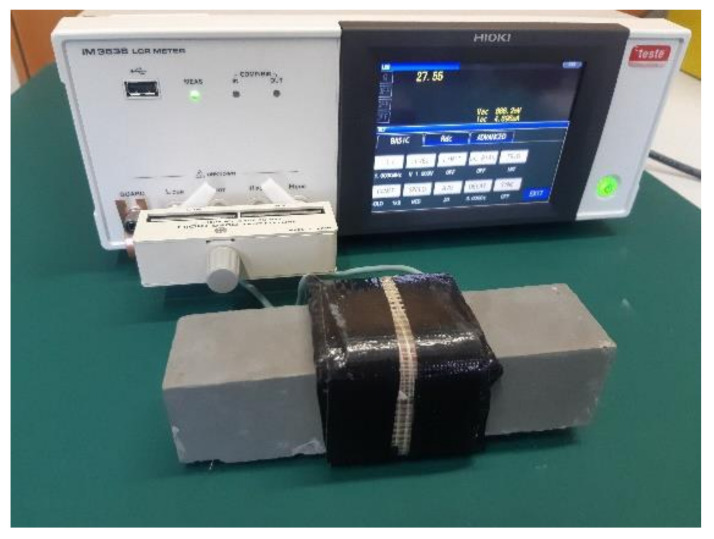
The setup for measuring 40 mm × 40 mm × 160 mm specimens.

**Figure 12:**
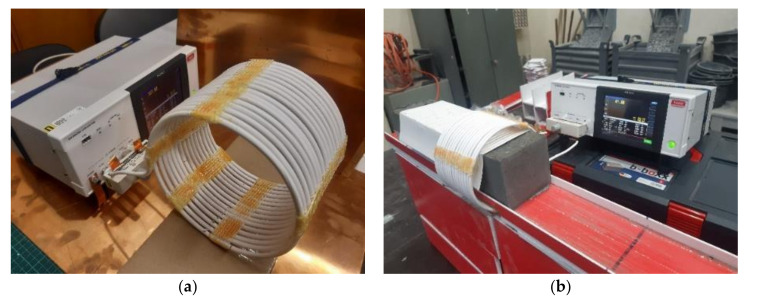
(**a**) The assembled larger measuring coil. (**b**) The setup for quality factor measurements for 100 mm × 100 mm × 400 mm specimens.

The measuring coil for the smaller specimens had a square cross-section and tightly encircled the measured material. The square cross-section coil was made from copper wire, with a cross-section area of 2.5 mm^2^ and 29 turns (Figure 11). The bigger specimens were measured using a circuit coil with a diameter of 155 mm and a length of 105 mm, made of 15 turns of copper wire, and with a cross-section area of 16 mm^2^ (Figure 12). All measurements of the quality factor *Q* were made with the impedance meter HIOKI IM3536 (HIOKI, Negano, Japan).

### 4.4. Initial Results and Quality Factor Measurements Evaluation

**Figure 13:**
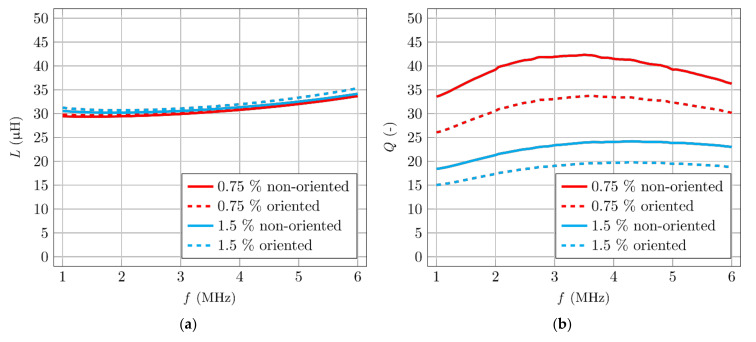
Comparison of the coil’s parameters between specimens with different volume and orientation of fibres. (**a**) Inductance measurements. (**b**) Quality factor measurements.

Initially, four smaller specimens were manufactured and tested: two with 0.75% and two with 1.5% fibre volume. One specimen from each sample had its fibres oriented by a magnetic field. Figure 13a shows the comparison between the dependency of inductance and Figure 13b shows the quality factor for different fibre volume and fibre orientation.

Inductance responds to changes better with a higher content of ferromagnetic particles. The quality factor has more convincing results, especially in the frequency domain around maximum losses. The value of the quality factor varies by tens of percentage points for specimens with differently oriented fibres. The measured induction differs only in the order of units of percentages for different types of orientation. Specifically, for the specimen with a 0.75% volume of fibre, the difference of quality factor between oriented and non-orientated specimens was 31%. Meanwhile, for the inductance, it was only 1.4%. Many authors [16,17,18,19,20,21] are using inductance as a non-destructive method for estimating fibre volume and orientation. Measuring changes in the quality factor is more sensitive compared to measuring changes in the inductance, and is, therefore, more accurate.

**Figure 14:**
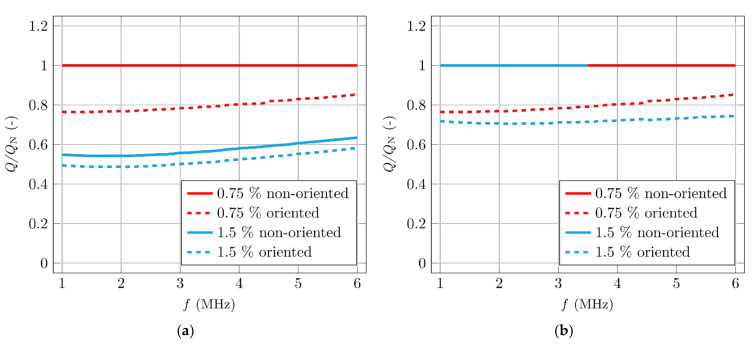
The dependency of the change of the quality factor. (**a**) Norm is a specimen with the lowest fibre content and the highest quality factor. (**b**) Norms are the non-oriented specimens.

The evaluation of the results only makes sense as a comparative measurement between two or more samples (using the same coil, the same impedance meter setting, etc.). A more suitable analysis may be obtained by standardizing the results by a norm. In Figure 14a, the specimen with the lowest volume of fibres that reached the highest quality factor value was selected as the norm, and the other results from Figure 13 were divided by this norm. Figure 14b has two norms used for the analysis of the results from Figure 13. Both norms are the results of the non-orientated specimens with different volumes of fibres, which are compared with oriented specimens. This comparison is more suitable for assessing the degree of orientation of the fibres. Figure 14 shows that this comparison would be more appropriate for specimens where only one parameter changes (volume of fibres).

**Figure 15:**
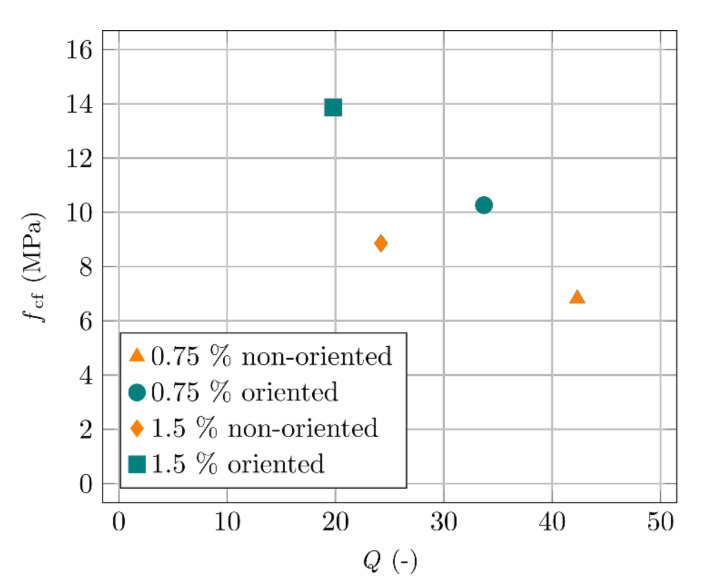
Comparison of the flexural strength and the quality factor.

The most important comparison is between the measured quality factor and the achieved mechanical properties of the specimens. Figure 15 shows the quality factors and flexural strengths (*f*_cf_) of the same aforementioned specimens, measured using a three-point bending experiment. Fibre orientation was further verified by CT scanning. Higher fibre content corresponds to a lower quality factor and higher flexural strength. Better fibre orientation corresponds to a lower quality factor and higher flexural strength.

### 4.5. Verification of the Results on a Larger Sample

Verification of the influence of the fibres’ orientation on the quality factor was performed for a large number of specimens for different magnetic field strengths and forms of the magnetic field (either DC or AC). For the same conditions, at least five specimens were prepared. These differences are not further described, as they are beyond the scope of this article. The main goal here is not to examine the influence of different inputs on the comparison results but to verify the possibility of using the measured values of the quality factor of the measuring coil as an indicator of the resulting mechanical parameters.

**Figure 16:**
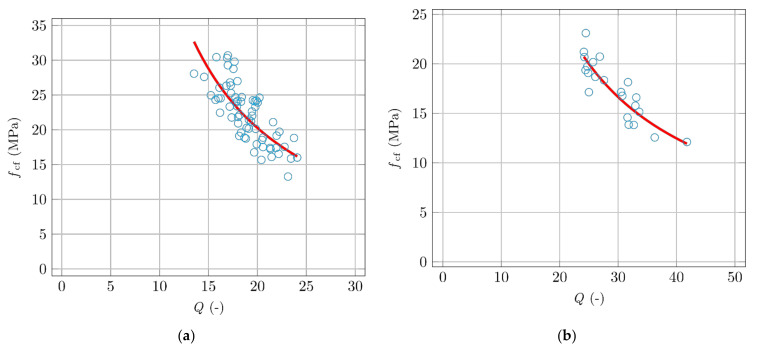
Comparison of the flexural strengths and measurements of the quality factor *Q*. (**a**) 40 mm × 40 mm × 160 mm specimens. (**b**) 100 mm × 100 mm × 400 mm specimens.

For evaluation, it is important to compare the corresponding measurements of specimens of the same size with the same measuring coil, and at the same volume of fibres in the mixture. Smaller specimens were exposed to a different form of magnetic field and strength. Bigger specimens were exposed to an AC magnetic field with an induction of 100 mT only. Specimens were not vibrated. The mixture for all specimens was the same, with 1.5% volume of fibres, Weidacon FM, and length of 13 mm. A three-point bending test was performed with the smaller specimens (Figure 16a) and a four-point bending test was performed with the larger specimens (Figure 16b).

Both graphs in Figure 16 show the possibility of a non-destructive method for estimating the degree of orientation of ferromagnetic fibres in the material (represented by the resulting flexural strength) by the quality factor *Q* of the measuring coil. The validity of the comparison is subjected to the above assumptions. It is necessary to compare specimens of the same size with the same fibre content, measured in the same measuring coil. A relationship between the measured quality factor and the resulting strength can be seen (lower fibre orientation in the desired direction, as well as lower fibre concentration, results in lower flexural strength).

### 4.6. Conclusions of the Quality Factor Measurements

By measuring the quality factor *Q* or its inverse value, it is possible to overview the number of fibres in the mixture with the same fibre orientation. It also makes it possible to evaluate fibre orientation for specimens with the same number of fibres. However, the measurement is comparative. It provides good agreement with the results of destructive tests. Nevertheless, it is only indicative of the fibre-related characteristics and cannot provide information about the concrete matrix (possible defects etc.), so it cannot replace standard destructive testing.

## 5. Conclusions and Further Outlook

The benefits of using ferromagnetic fibres to improve the mechanical properties of concrete can be further improved by achieving the preferred fibre orientation using a magnetic field. The resulting material has anisotropic properties, which can improve the characteristics in the desired direction or reduce the required amount of added fibres and achieve economic savings. The fibres’ ferromagnetic properties are crucial, so that it is possible to orient them in the cement matrix using a magnetic field. However, it is also necessary to be able to determine whether this orientation is successful and to what degree. That is the main purpose of this non-destructive principle. It has been shown that it is sensitive enough for these purposes.

The paper points out the fact that the magnetic properties of the materials used as fibres are nonlinear not only at a sufficient magnetic field strength (when the material is practically saturated) but also at low magnetic field intensities. The material behaves as a “very bad” ferromagnetic material in the magnetic field with low intensity. The magnetic induction, in that case, is not sufficient, and the resulting force acting on the fibre is significantly lower compared to, for example, the quality material used for electro-magnets, with linear growth of the primary magnetization curve.

Magnetic properties of the fibres also allow for measuring the number of ferromagnetic fibres present and their orientation. This paper shows the suitability of such techniques for comparative measurements between different specimens, and highlights the advantages of measuring changes in the quality factor over measuring other parameters, such as changes in the inductance, and the importance of choosing the appropriate frequency range. The non-destructive method based on measuring the quality factor *Q* to estimate fibre volume and orientation is a promising and elegant solution to ensure quality in future serial production. Measuring coils could be scaled up for full-scale structural elements. However, the best results would be achieved with coils specifically designed for the geometry of said elements. This could be feasible in, for example, a prefabrication industry for various concrete columns, beams, etc.

## Data Availability

The data presented in this study are available on request from the corresponding author.
